# Efficient expansion of global protected areas requires simultaneous planning for species and ecosystems

**DOI:** 10.1098/rsos.150107

**Published:** 2015-04-29

**Authors:** Tal Polak, James E. M. Watson, Richard A. Fuller, Liana N. Joseph, Tara G. Martin, Hugh P. Possingham, Oscar Venter, Josie Carwardine

**Affiliations:** 1 School of Biological Sciences, University of Queensland, St Lucia, Queensland 4072, Australia; 2 School of Geography, Planning and Environmental Management, University of Queensland, St Lucia, Queensland 4072, Australia; 3 Global Conservation Program, Wildlife Conservation Society, Bronx, NY 10460, USA; 4 CSIRO Land and Water, PO Box 2583, Brisbane, Queensland 4001, Australia; 5 Department of Life Sciences, Imperial College London, Silwood Park, Ascot SL5 7PY, Berkshire, UK; 6 Centre for Tropical Environmental and Sustainability Science, James Cook University, Cairns, Queensland 4878, Australia

**Keywords:** ecosystem-based targets, surrogacy, spatial prioritization, geographical range size, adequacy, representation, CBD Aichi targets

## Abstract

The Convention on Biological Diversity (CBD)'s strategic plan advocates the use of environmental surrogates, such as ecosystems, as a basis for planning where new protected areas should be placed. However, the efficiency and effectiveness of this ecosystem-based planning approach to adequately capture threatened species in protected area networks is unknown. We tested the application of this approach in Australia according to the nation's CBD-inspired goals for expansion of the national protected area system. We set targets for ecosystems (10% of the extent of each ecosystem) and threatened species (variable extents based on persistence requirements for each species) and then measured the total land area required and opportunity cost of meeting those targets independently, sequentially and simultaneously. We discover that an ecosystem-based approach will not ensure the adequate representation of threatened species in protected areas. Planning simultaneously for species and ecosystem targets delivered the most efficient outcomes for both sets of targets, while planning first for ecosystems and then filling the gaps to meet species targets was the most inefficient conservation strategy. Our analysis highlights the pitfalls of pursuing goals for species and ecosystems non-cooperatively and has significant implications for nations aiming to meet their CBD mandated protected area obligations.

## 2. Introduction

Protected areas are pivotal for conserving both ecosystems and threatened species [1]. While the persistence of biodiversity often requires a suite of management strategies, protected areas provide a buffer from a myriad of threatening processes [2,3]. Gap analyses of the current global coverage of protected areas (approx. 12% of Earth's land surface) highlight that many ecosystems and most threatened species are not well represented [4–9]. In most countries, a lack of systematic planning has given rise to significant biases in the location of protected areas [10,11]. Typically conservation land was selected in locations that were not required for other, more lucrative, land uses [11]. Systematic conservation planning approaches have sought to redress these biases by using spatial data on species distributions and ecosystems to prioritize locations for new protected areas [12]. The expansion of current protected area networks has the potential to overcome these biases and improve ecosystem protection and the survival of threatened species populations, helping to avoid biodiversity loss and species extinctions [5,6,13,14].

Over the past decade, there has been a major shift towards ecosystem-based planning for the expansion of protected area networks [15–18]. Ecosystem-based targets use environmental surrogates at various scales (e.g. bioregions and ecoregions) with the intention of efficiently representing biodiversity as a whole, including species and processes [15,19]. Ecosystem-based planning is used as an approach to avoid the often challenging need for unbiased, high-resolution information on the spatial distributions of species [20–22]. Much of the existing species data have sparse or patchy distributions biased by survey effort, causing species-driven conservation planning to fail in representing biodiversity as a whole [23]. Ecosystem data are typically spatially contiguous and coarser than species data, thereby providing more flexibility for locating new protected areas and more options for avoiding conflicting land uses [8].

Critics of ecosystem-based targets argue that they are too coarse to effectively represent biodiversity, allowing many species to fall through the cracks [1,20,24,25]. For example, both Araújo *et al*. [24] and Lombard *et al*. [22] found that environmental surrogates perform relatively well for plants but fail to adequately represent vertebrates, while Rodrigues & Brooks [1] found that environmental surrogates were poor at representing species. Further, the effectiveness of environmental surrogates decreases for species that are rare, have patchy or historically driven distribution and/or are data deficient, which is the case for many threatened species [21,22].

The global goals stated within the Convention on Biological Diversity (CBD) strategic plans in both 2004 and 2010 are a primary reason for the shift from species-based to ecosystem-based planning for future protected area acquisitions [16]. Specifically, in the 2010 CBD's Aichi target 11, there is a clear goal to conserve 17% of terrestrial and inland waters and 10% of marine and coastal ecosystems [16]. The CBD states that networks should be ecologically representative, but gives no specific stipulation for how much habitat for threatened species should be protected [16]. The achievement of threatened species protection under Aichi target 11 is therefore open to interpretation by nations considering the expansion of their protected area estate.

There are 195 parties that are part of the CBD treaty, each with the opportunity to translate these goals to a national level in ways that may result in various outcomes for biodiversity represented in protected areas. For example, ecosystems are the base unit of the South Africa National Protected Area Expansion Strategy, which plans to achieve the 17% representation by targeting different proportions of ecosystems depending upon their diversity and protection requirements [18]. Similarly, Australia's National Reserve System's (NRS's) primary goal at the time of research was to protect 10% of each of its 85 bioregions [26] by representing at least 80% of the different types of ecosystems within each bioregion by 2015, with a secondary goal to represent core areas for threatened species by 2030 [26]. This plan is an example of a coarse/fine filter approach, which advocates planning for ecosystems and then filling the gaps for species [27,28], which is also applied in North America [28,29].

The use of ecosystem-based targets, both in global protected area guidelines and country-level protected area expansion policy, has occurred in the absence of scientific analyses on how efficiently the ecosystem-based surrogates represent threatened species. While several studies [1,30,31] have examined the comparative benefits of both approaches, none have investigated their efficiency in protecting both species and ecosystems in the context of the CBD's strategic plan and country-level priority setting. Full implementation of the 2010 CBD strategic plan across all signatory countries is likely to take considerable time and resources, but would represent one of the greatest expansions of the global protected area estate in modern times [8]. An improved understanding of how an ecosystem-based approach is likely to impact conservation outcomes is therefore timely and will assist countries in translating the CBD goals into protected area expansion that efficiently and effectively conserves threatened species as well as ecosystems.

Here, we conduct a novel assessment of the impact of ecosystem-based planning and the global CBD goals on protected area outcomes for threatened species at the country level upon which they are implemented. We assess the potential for efficiency and effectiveness in threatened species and ecosystem coverage in the expanding protected area network across Australia, arguably the first country to fully embrace a systematic planning approach using ecosystem-based targets to design the protected area estate [26,32,33]. A recent continent-wide analysis by Watson *et al*. [34] found that despite this relatively systematic approach, threatened species coverage in the protected area estate (which covered 11.6% of the terrestrial surface) is still inadequate, with approximately 12% of threatened species completely absent from the protected area network. The Commonwealth of Australia's plan to expand their reserve system (known as the NRS [26,35]) follows the guidelines and ecosystem-based approach suggested by the CBD.

Specifically, we investigate (i) how well threatened species are likely to be captured in the resultant protected area network if Australia aims to meet its 10% targets for all ecosystems most efficiently (as current policies suggest) by minimizing the area required; (ii) how well ecosystem-based targets are likely to be met if Australia's protected area network is designed to meet targets for threatened species only; and finally (iii) the efficiency and effectiveness of planning for both sets of targets simultaneously versus sequentially (e.g. meeting ecosystem targets first and species targets later or vice versa). We define efficiency as the amount of area required to meet a given set of targets, and effectiveness as the level of representation of a target in a given protected area network.

## 3. Material and methods

### 3.1 Ecosystem spatial data and targets

At the time of this research, Australia was divided into 85 bioregions, hereafter ‘ecosystems’, based on the Interim Biogeographic Regionalization of Australia (IBRA bioregions [33], v. 6.1), at a spatial resolution of approximately 10 km^2^. These ecosystems were derived by compiling geographical information on continental scale gradients and patterns in climate, substrate, landform, vegetation and fauna, and each bioregion is considered a distinct ecologically and geographically defined area [35]. The Commonwealth of Australia [26,35] has set a target of at least 10% representation in each ecosystem in the protected area estate for the NRS, which is the ecosystem target adopted in this study.

### 3.2 Threatened species data and targets

We considered distributions of 1320 species from the total of 1737 species listed under the Environmental Protection and Biodiversity Conservation Act. We used maps of species' distributions at a resolution of approximately 10 km^2^, developed for extant terrestrial and freshwater threatened species available in the Species of National Environmental Significance database [36]. We excluded 95 extinct species and 367 species that are marine, freshwater or migratory, or whose distributions are only estimated with low certainty. The species we considered, hereafter referred to as ‘threatened species’ are listed as Critically Endangered, Endangered or Vulnerable [36] (note that the definitions of these categories as applied within Australia differ slightly from those employed globally by the IUCN Red List, and also that there are species on the IUCN Red List that are not listed nationally, and vice versa).

Following Watson *et al*. [34] and building on a method developed by Rodrigues *et al*. [13] and Kark *et al*. [37], we set a series of adequacy targets for these 1320 threatened species based on geographical range size and level of endangerment. This method develops area-based targets that scale with geographical range size, requiring species with smaller ranges to be increasingly well protected [5,13,38–40]. A target of complete coverage (i.e. 100% of remaining extent) by protected areas was set for those species considered Critically Endangered and those species with a geographical range size of less than 1000 km^2^. Conversely, for those species with large range sizes (more than 10 000 km^2^), the target was set to cover 10% of current range. For species with geographical ranges of intermediate size (between 1000 and 10 000 km^2^), the target was linearly interpolated between these two extremes, with decreasing representation targets (smaller percentage of their range) set for species with larger range sizes (electronic supplementary material, figure S1).

### 3.3 Spatial prioritization analyses

We determined the amount of each of the 85 ecosystems and 1307 threatened species already covered by the current protected area estate by intersecting the ecosystem maps and threatened species distribution maps with the map of the Australian protected area estate [41] (this includes IUCN management categories I–VI). For both ecosystems and species, we masked out distributions that occurred in cleared areas (i.e. are not potential for conservation). For some species, the area of remaining available intact habitat was smaller than their set target. In such cases, we reduced the target for these species to represent 100% of remaining available intact habitat. Thirteen of our 1320 species had none of their distribution within areas that were considered intact and available for conservation, and were counted as gap species and their targets were set to zero. This left 1307 species as our threatened species target set.

We created a planning unit layer of 10 × 10 km grid cells covering Australia, which was the smallest resolution computationally feasible and approximately matches the scale of the maps of threatened species [34] and ecosystems [42]. We intersected the planning unit layer with the protected area layer, such that each existing protected area was a separate planning unit. We determined the amount of each species and ecosystem type in each planning unit based on their spatial overlap.

We used the systematic conservation planning software Marxan [43] to identify solutions for the expansion of Australia's protected area network to meet the above targets for ecosystems and threatened species coverage. Marxan uses a simulated annealing algorithm to select multiple alternative sets of areas that meet pre-specified biodiversity targets while minimizing overall cost [43]. It has been used for identifying proposed conservation areas in Australia and throughout the world (e.g. [44–46]). When investigating spatial options for expansion, we locked in the current protected area estate and assumed that targets for all species and ecosystems were of equal importance to meet. We set the cost of each planning unit as the total area potentially suitable for conservation within the planning unit, i.e. we assumed that only areas of native vegetation would be suitable for inclusion in the protected area estate, and we used area is a universal surrogate for the costs of protected area management [43].

We used Marxan to identify 500 solutions for each of five scenarios (table 1). First, we identified the additional area required to protect all the ecosystem-based targets (by being added to the current network) and assessed how well the selected network covered the adequacy targets for the 1307 threatened species (table 1, scenario 1). Next, we determined the minimum amount of newly protected land needed to meet the range-based targets for threatened species and assessed how well this solution met the representation targets set for ecosystems (table 1, scenario 2). In our third and fourth scenarios (table 1), we investigated how much additional land area would be required to be added to the network formed in scenarios 1 and 2 to achieve all targets for both species and ecosystems, i.e. achieving 100% of all targets in a stepwise way. Lastly, for scenario five, we established the minimum amount of land needed to create a protected area network for both ecosystems and species targets when planning simultaneously (table 1).

**Table 1.** Scenario results: area of proposed protected areas and amount of species and ecosystem targets that are adequately protected.

	current situation	scenario 1 achieving 10% ecosystem targets	scenario 2 achieving threatened species coverage targets	scenario 3 achieving 10% ecosystem targets then adding species targets	scenario 4 achieving threatened species coverage targets then adding 10% ecosystem targets	scenario 5 achieving both threatened species and ecosystem targets simultaneously
land covered in protected areas in ha (% of Australia)	89 115 652 (11.59%)	118 629 670 (15.43%)	143 988 700 (18.73%)	168 574 630 (21.93%)	161 689 810 (21.03%)	161 191 100 (20.96%)
threatened species coverage						
no. species adequately protected (% of total species)	284 (21.5%)	323 (24.7%)	1307 (100%)	1307 (100%)	1307 (100%)	1307 (100%)
average proportion^a^ of species target met	47.8%	52.2%	99.9%	99.9%	99.9%	99.9%
ecosystems coverage						
no. ecosystems with 10% coverage	48 (56.5%)	85 (100%)	60 (70.6%)	85 (100%)	85 (100%)	85 (100%)
average proportion^a^ of 10% ecosystems coverage achieved	72.6%	100%	85.1%	100%	100%	100%

^a^Some features had more than 100% of their target met but for the analysis reported in this table, we only allowed a maximum of 100% coverage.

All five scenarios were evaluated by: (i) the total number and percentage of targets that were fully met; and (ii) the average proportion of coverage for each set of targets (species and ecosystems) in the top solution for each scenario (coverage was calculated per target, and if more than 100% of a target was met, it was capped at 100% when averaging across all targets in the set). We compared the dissimilarity between scenarios (1–5) using Jaccard distances for both the added protected areas only and the entire network including the existing protected areas (electronic supplementary material, table S1).

We also investigated the sensitivity of our results to: (i) the measure of cost we used (area) by comparing with a cost based on forgone agricultural opportunities [47]; and (ii) the possible impact of the reserve expansion being driven by current location of protected areas, by testing a scenario where protected areas were not locked in. First, we re-ran scenarios 1–5 (table 1) with the cost of protecting a planning unit based on its current agricultural profitability, instead of its area (higher profitability values represent higher opportunity costs). We used a GIS agricultural profit map ($ ha^−1^) from Marinoni *et al*. [47] to calculate a per planning unit annual profitability value in $ ha^−1^ and multiplied it by the area of the planning unit potentially suitable for conservation, and used this value as the planning unit cost. Negative values were rounded to zero and a transaction cost of $10 000 was added proportionally to the planning size as the minimum land value [44]. Second, we re-ran the analysis without locking in existing protected areas, thus capturing a scenario where no fixed current protected areas network exists and all non-cleared areas are available for conservation.

## 4. Results

There is a large degree of variation in the coverage of ecosystems (bioregions) and threatened species in the current protected area network (table 1). Forty-eight (56.5%) ecosystems have achieved their target of 10% protected; however, some ecosystems are poorly represented, and on average ecosystems have attained 72.6% of their target level of protection. The protected area estate is performing worse for threatened species, with only 284 (21.5%) threatened species reaching their range-based target. On average, across all species, 47.3% of the target area is covered in the current network (table 1).

**Figure 1.**
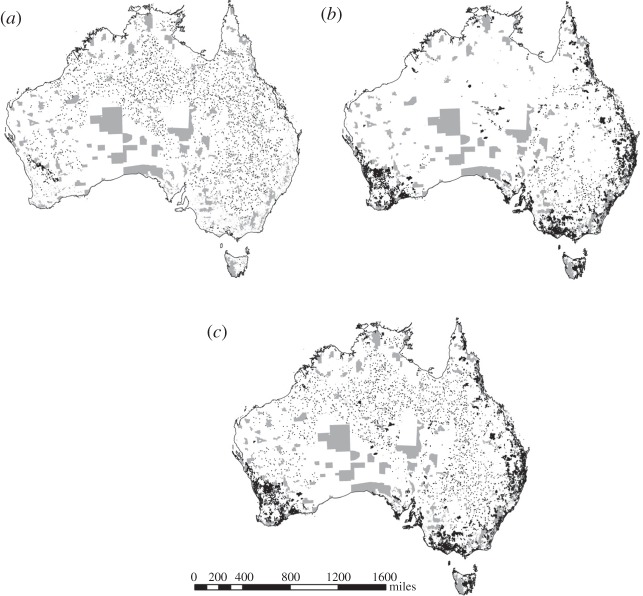
Spatial distribution of proposed protected areas and existing protected areas for each of the planning scenarios. Grey areas represent the current protected areas; black areas represent the proposed additional protected areas for each scenario's best solution. (*a*) Achieving 10% ecosystem targets; (*b*) achieving threatened species coverage targets; and (*c*) achieving both threatened species and ecosystem targets simultaneously.

We found that a minimum of 29.5 million hectares must be added to the existing protected area network to achieve 10% representation of each ecosystem. The solution with the sole target of achieving 10% of each ecosystem in the smallest amount of area requires approximately 15.4% (table 1) of terrestrial Australia to be in protected areas (figure 1*a*). One of the main reasons that the required national-level coverage is greater than the 10% target is because some ecosystems, mostly arid ecosystems [48], have been protected to a level above 10%. Another reason is that many planning units are selected to meet targets for ecosystems that only occur in a small portion of the unit. Expansion aimed solely at increasing ecosystem representation would incidentally increase the number of threatened species adequately captured by 3.2% and increase the average proportion of adequacy targets met across all species from 47.3% up to 52.2% (table 1).

Expanding the current protected area network to represent all threatened species adequately without considering ecosystem targets would require an additional 54.9 million hectares. This equates to a protected area system that is approximately 144 million hectares in size (or 18.7% of Australia; table 1 and figure 1*b*). In this scenario, the number of ecosystems protected to a 10% level would increase from 48 to 60.

**Figure 2.**
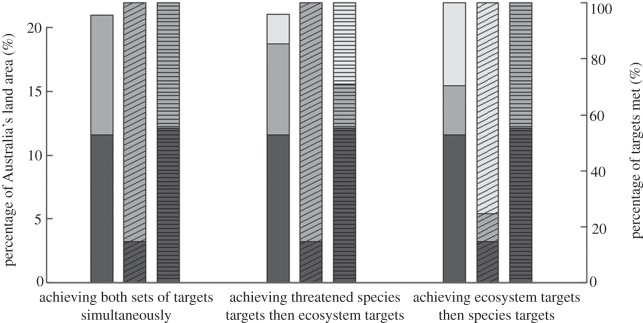
Comparing the efficiency of the two stepwise scenarios and the simultaneous scenario (scenarios 3–5): from the existing protected areas network (dark grey) to the first step of protected areas selection (mid-grey), to the second step (light grey) of filling the gaps for the opposite target-type (ecosystem or species). The plain bars (left *y*-axis) show the increase in land area (as % of Australia's land area). The diagonally striped bars represent percentage of species targets met (right *y*-axis). The horizontally striped bars represent percentage of ecosystem-based targets met (right *y*-axis).

If the protected area network is expanded to meet ecosystems and threatened species targets simultaneously, an additional 72 million hectares would require protection (approx. 21.0% of Australia's land surface; figure 1*c* and figure 2). Planning to meet these same goals sequentially, starting with ecosystems targets and then adding areas to meet threatened species targets, would require 79.5 million hectares (21.9% of the land; table 1 and figure 2) to be added to the existing network, which is approximately 7 million hectares more than the most efficient scenario that integrated these targets. Planning sequentially, starting with threatened species targets and then adding areas to achieve the 10% ecosystems representation target, will require a similar amount of land as planning simultaneously (table 1 and figure 2).

There are substantial differences in the spatial configuration of the protected areas network expansion between the five scenarios (electronic supplementary material, table S1). The majority of the difference lies between scenarios 1 and 2 (selection for ecosystems or selection for species, figure 1*a,b*, Jaccard dissimilarity distance of added protected areas *d*_*j*_ = 0.982). When planning for ecosystems only (figure 1*a*), we get a map of protected areas which are arranged relatively evenly throughout Australia as a result of the nature of the ecosystems, which are large non-overlapping spatial features. When planning for threatened species (figure 1*b*), the resulting network is concentrated around the coastline, reflecting the fact that species distributions are usually small dynamic units affected by internal and external processes and reflect past land-use changes [49]. Larger protected areas might be more effective in promoting persistence for biodiversity and might be more robust to climate change [50,51]. However, a species-based approach is likely to protect more areas that are threatened by habitat loss and valuable for recreational use by society [52].

The same pattern of cost-effectiveness holds when using agricultural profits as the cost layer instead of land area, indicating that our analysis was robust to using either of these costs surrogates (electronic supplementary material, figure S2). Planning for ecosystems only marginally improves protection for threatened species and filling the gaps to reach 100% of species targets delivered the most costly scenario (electronic supplementary material, table S2). Planning for both targets simultaneously again proved the most cost-effective approach, followed by sequentially planning first according to species targets and then adding areas to achieve ecosystem targets (electronic supplementary material, table S2).

When performing the same analyses but assuming that no current protected area network exists, scenarios follow the same overall pattern, with simultaneous planning (scenario 5) being the most cost-effective closely followed by sequential planning starting with species (scenario 4). Again, sequentially planning starting with ecosystems (scenario 3) was the least cost-effective option (electronic supplementary material, table S3). Planning according to ecosystems only (scenario 1) required less area than the current protected area network but only protected 9% of the species while planning according to species achieved 60% of the ecosystems targets (electronic supplementary material, table S3).

## 5. Discussion

If implemented by signatory nations, the CBD 2010 strategic plan will lead to the largest increase in global protected area establishment in history [7,8]. Importantly, national protected area plans adopted by governments often follow the global CBD guidelines, which have explicitly promoted an ecosystem-based approach to achieve the expansion [16,53]. We tested whether an ecosystem-based approach, by itself, would be effective in adequately conserving threatened species, using Australia as a case study. We found that prioritizing future protected areas based on representing the 85 major ecosystems is likely to fall well short of capturing adequate amounts of many (approx. 75%) threatened species' ranges.

We found that planning for the expansion of protected area networks to meet targets for ecosystems and species at the same time will achieve both sets of targets with fewer resources and less land. This result contrasts with the stepwise coarse/fine filter approach, which we show is likely to be a less efficient way to achieve targets for both species and ecosystems together. Our results concur with other analyses which show that if the goal is to protect species and ecosystems, a dual approach is most effective [21,22,49]. Simultaneous planning is most efficient because the planning units that collectively meet both goals most efficiently can be identified, avoiding the selection of planning units that become redundant once a secondary goal is added.

The efficiencies gained by simultaneous planning on a continent the size of Australia are modest, but could become enormous once multiplied up to a global scale. Assuming the same patterns hold, the efficiencies gained by planning for threatened species and ecosystems together across the world would equal an area the size of a third of all EU countries. Our results have significant implications for how nations should interpret the CBD strategic plan. Implementation of the ecosystem-based targets alone is likely to mean future protected areas will not be optimal to meet each country's commitment to protecting threatened species, nor the overall aim of the CBD. Considering the biodiversity crisis most nations currently face [54] and the limited amount available for conservation [55], future acquisitions of protected areas need to be efficient in achieving ecosystem and threatened species representation.

The disparity between protected area network expansion for threatened species targets versus ecosystem-based targets is due to the differences in the spatial resolution of the two types of features. Ecosystems are large and non-overlapping, permitting flexibility in which planning units are selected for conservation and promoting a spatially even spread of protected areas. Alternatively, species distributions are often smaller, can overlap, are spatially aggregated and reflect land-use history [49,52]. As such, the areas still available to these species are spatially skewed and usually small compared with the large and widely distributed ecosystems [49,56]. While many nations do not have extensive spatial data on their threatened species, the IUCN Red List assessments make this freely available when it does exist [57].

We do not attempt to present a future plan for Australia's protected area network, which would entail the inclusion of further social, economic and biological considerations. We assume all areas are available for protected area expansion, but in reality factors such as opportunities for landholder engagement, public accessibility and feasibility would impact on this availability [39,58,59]. Further, while a well-managed, well-placed protected area network provides a key component required to facilitate the persistence and recovery of threatened species [52,60–62], many threatened species require a more intensive management programme than gazetting of protected areas alone [63,64]. The full costs of protecting and managing areas include the opportunity costs of not developing a site, direct costs such as infrastructure, maintenance and salaries, and the costs of planning and implementing management programmes [65,66].

Additional ecological considerations required in a real-world protected area expansion task also include the consideration of species distributions under climate change, minimum protected area size, and connectivity and corridors, which may be important for improving the likelihood that species will persist in reserves over the long term. Future research will need to consider the dynamic nature of threats such as land-use change and climate change, presenting a need to assess both species and ecosystem range shift to these changes. Moreover, given the role of biodiversity-driven ecosystem services such as pollination, pest control and recreation [67], it may be important for real-world planning to consider ecosystem services. Data and targets for threatened species, ecosystems and protected areas are regularly updated but the minor changes that have recently occurred are not likely to affect the conclusions of our analyses [36,68,69].

We found that expanding the protected area network to meet the targets used in this study would result in forfeiting (or shifting the locations for generating) almost 5 billion dollars in annual potential agricultural profit. Due to the simplifications we made in our analysis, it is likely that a real-world comprehensive protected area network in Australia would require a larger total area being needed to meet ecosystem and species targets, as the most ‘efficient’ options may not be available nor sufficient to ensure species persistence. We also note that protected areas can provide alternative sources of income by creating jobs, helping to develop rural areas and tourism revenues and providing benefits for human health and wellbeing while helping to protect the intrinsic values of nature [70]. Regardless of the cost and size of the resultant protected area estate, an efficiency approach such as the one we present serves to minimize the costs of providing these benefits to society.

Biodiversity loss is a global problem. However, the expansion of protected area networks is typically planned at a national level. In countries such as Australia, where ecosystem and species databases exist, planning for both ecosystems and species can occur simultaneously to deliver the most efficient solutions. Countries with protected area expansion plans inspired by interpretations of the CBD guidelines [17,18,26] need to consider the best available data on both species and ecosystems if both are to be efficiently protected.

## Supplementary Material

Fig S1: Target calculation diagram provided from unpublished data related to Watson and colleagues. For species with a range size smaller than 1000 km2 a target of 100% of their range was set (upper horizontal line). For species with a range size larger than 10,000 km2 a target of 10% of their range was set (lower horizontal line). For species with an intermediate range size of 1,000-10,000km2 a target was interpolated between the upper and lower values (along the diagonal line)

## Supplementary Material

Fig. S2: Comparison of land area required for the protected area network for each scenario (represented in percentages of Australia's land area) when planning to minimize land area (dark grey); when planning to minimize agricultural losses (light grey) and when current protected areas are ignored (i.e. not locked in to the solution) (black).

## Supplementary Material

Table S1- Comparing between scenarios using Jaccard Dissimilarity index.

## Supplementary Material

Table S2- Scenario results: Area of proposed protected areas and amount of species and ecosystem targets that are adequately protected - Agricultural costs

## Supplementary Material

Table S3- Scenario results: Area of proposed protected areas and amount of species and ecosystem targets that are adequately protected without an existing protected areas network

## Data accessibility

The databases supporting our study are freely available on the web and the appropriate references are cited.
