# Supplementary material for: Efficient expansion of global protected areas requires simultaneous planning for species and ecosystems
Source: R Soc Open Sci. 2015 Apr 29;2(4):150107. doi: 10.1098/rsos.150107 (PMC4448872; doi:10.1098/rsos.150107)
Supplement: Fig S1: Target calculation diagram provided from unpublished data related to Watson and colleagues. For species with a range size smaller than 1000 km2 a target of 100% of their range was set (upper horizontal line). For species with a range size larger than 10,000 km2 a target of 10% of their range [file rsos150107supp1.docx]

# Fig S1

Fig S1: Target calculation diagram provided from unpublished data related to Watson and colleagues [34]. For species with a range size smaller than 1000 km^2^ a target of 100% of their range was set (upper horizontal line). For species with a range size larger than 10,000 km^2^ a target of 10% of their range was set (lower horizontal line). For species with an intermediate range size of 1,000-10,000km^2^ a target was interpolated between the upper and lower values (along the diagonal line)
