# Supplementary material for: Efficient expansion of global protected areas requires simultaneous planning for species and ecosystems
Source: R Soc Open Sci. 2015 Apr 29;2(4):150107. doi: 10.1098/rsos.150107 (PMC4448872; doi:10.1098/rsos.150107)
Supplement: Fig. S2: Comparison of land area required for the protected area network for each scenario (represented in percentages of Australia's land area) when planning to minimize land area (dark grey); when planning to minimize agricultural losses (light grey) and when current protected areas are ignored (i [file rsos150107supp2.docx]

# Fig. S2

Fig. S2: Comparison of land area required for the protected area network for each scenario (represented in percentages of Australia’s land area) when planning to minimize land area (dark grey); when planning to minimize agricultural losses (light grey) and when current protected areas are ignored (i.e. not locked in to the solution) (black).
