# Supplementary material for: Efficient expansion of global protected areas requires simultaneous planning for species and ecosystems
Source: R Soc Open Sci. 2015 Apr 29;2(4):150107. doi: 10.1098/rsos.150107 (PMC4448872; doi:10.1098/rsos.150107)
Supplement: Table S1- Comparing between scenarios using Jaccard Dissimilarity index. [file rsos150107supp3.docx]

# Table S1- Comparing between scenarios using Jaccard Dissimilarity index. The numbers above the diagonal represent the distances between the scenarios for the new protected areas only and the numbers below the diagonal represent the distances between the scenarios for the entire network, including the existing protected areas.

|  | Scenario 1  Achieving 10% ecosystem targets | Scenario 2  Achieving threatened species coverage targets | Scenario 3  Achieving 10% ecosystem targets then achieving species targets | Scenario 4  Achieving threatened species coverage targets then Achieving 10% ecosystem targets | Scenario 5  Achieving both threatened species and ecosystem targets simultaneously |
| --- | --- | --- | --- | --- | --- |
| Scenario 1  Achieving 10% ecosystem targets | 0 | 0.98 | 0.71 | 0.95 | 0.95 |
| Scenario 2  Achieving threatened species coverage targets | 0.33 | 0 | 0.56 | 0.26 | 0.38 |
| Scenario 3  Achieving 10% ecosystem target then achieving species targets | 0.27 | 0.22 | 0 | 0.55 | 0.56 |
| Scenario 4  Achieving threatened species coverage target then Achieving 10% ecosystem targets | 0.35 | 0.08 | 0.23 | 0 | 0.45 |
| Scenario 5  Achieving both threatened species and ecosystem targets simultaneously | 0.35 | 0.12 | 0.23 | 0.16 | 0 |
|  |  |  |  |  |  |
