# Supplementary material for: Efficient expansion of global protected areas requires simultaneous planning for species and ecosystems
Source: R Soc Open Sci. 2015 Apr 29;2(4):150107. doi: 10.1098/rsos.150107 (PMC4448872; doi:10.1098/rsos.150107)
Supplement: Table S2- Scenario results: Area of proposed protected areas and amount of species and ecosystem targets that are adequately protected - Agricultural costs [file rsos150107supp4.docx]

# Table S2- Scenario results: Area of proposed protected areas and amount of species and ecosystem targets that are adequately protected – Agricultural costs

|  | Current situation | Scenario 1  Achieving 10% ecosystem targets | Scenario 2  Achieving threatened species coverage targets | Scenario 3  Achieving 10% ecosystem targets then achieving species targets | Scenario 4  Achieving threatened species coverage targets then Achieving 10% ecosystem targets | Scenario 5  Achieving both threatened species and ecosystem targets simultaneously |
| --- | --- | --- | --- | --- | --- | --- |
|  |  |  |  |  |  |  |
| Cost in agricultural profits ($) | 399,155,453 | 433,157,482 | 4,866,346,856 | 4,892,078,578 | 4,884,714,984 | 4,882,251,716 |
| Land Area (ha) | 89,115,652 | 119,686,896 | 146,548,225 | 171,563,300 | 163,991,556 | 163,108,658 |
| *Threatened species coverage* |  |  |  |  |  |  |
| Number of species adequately protected  (% of total species) | 284 (21.5%) | 331 (25.3%) | 1307 (100%) | 1307 (100%) | 1307 (100%) | 1307 (100%) |
| Average proportion^^^ of species target met | 47.8% | 52.5% | 99.8% | 99.8% | 99.8% | 99.8% |
| *Ecosystems coverage* |  |  |  |  |  |  |
| Number of ecosystems with 10% coverage | 48 (56.5%) | 85 (100%) | 61 (71.8%) | 85 (100%) | 85 (100%) | 85 (100%) |
| Average proportion^^^ of 10% ecosystems coverage achieved | 72.6% | 100% | 85.4% | 100% | 100% | 100% |
|  |  |  |  |  |  |  |

^^^Some features had more than 100% of their target met but for the analysis reported in this table we only allowed a maximum of 100% coverage.
