# Supplementary material for: Efficient expansion of global protected areas requires simultaneous planning for species and ecosystems
Source: R Soc Open Sci. 2015 Apr 29;2(4):150107. doi: 10.1098/rsos.150107 (PMC4448872; doi:10.1098/rsos.150107)
Supplement: Table S3- Scenario results: Area of proposed protected areas and amount of species and ecosystem targets that are adequately protected without an existing protected areas network [file rsos150107supp5.docx]

# Table S3- Scenario results: Area of proposed protected areas and amount of species and ecosystem targets that are adequately protected without an existing protected areas network

|  | Scenario 1  Achieving 10% ecosystem targets | Scenario 2  Achieving threatened species coverage targets | Scenario 3  Achieving 10% ecosystem targets then achieving species targets | Scenario 4  Achieving threatened species coverage targets then Achieving 10% ecosystem targets | Scenario 5  Achieving both threatened species and ecosystem targets simultaneously |
| --- | --- | --- | --- | --- | --- |
|  |  |  |  |  |  |
| Land covered in protected areas in ha  (% of Australia) | 79,749,870 (10.4%) | 101,807,550  (13.2%) | 136,600,920  (17.7%) | 120,000,460  (15.6%) | 117,565,970  (15.3%) |
| Efficiency from expanding current PA^$^ in ha | 38,879,800 | 42,181,150 | 32,572,710 | 41,689,350 | 43,553,130 |
| *Threatened species coverage* |  |  |  |  |  |
| Number of species adequately protected  (% of total species) | 120 (9.2%) | 1307 (100%) | 1307 (100%) | 1307 (100%) | 1307 (100%) |
| Average proportion^^^ of species target met | 28.6% | 99.9% | 99.9% | 99.9% | 99.9% |
| *Ecosystems coverage* |  |  |  |  |  |
| Number of ecosystems with 10% coverage | 85 (100%) | 51 (60%) | 85 (100%) | 85 (100%) | 85 (100%) |
| Average proportion^^^ of 10% ecosystems coverage achieved | 100% | 84% | 100% | 100% | 100% |
|  |  |  |  |  |  |

^^^Some features had more than 100% of their target met but for the analysis reported in this table we only allowed a maximum of 100% coverage.
^$^Protected area network
